# Survival of surface bacteriophages and their hosts in *in situ* deep-sea environments

**DOI:** 10.1128/spectrum.04534-22

**Published:** 2023-12-05

**Authors:** Wei Wei, Yuan Tian, Lanlan Cai, Yongle Xu, Xilin Xiao, Qiong Wang, Haowen Wang, Chunming Dong, Zongze Shao, Nianzhi Jiao, Rui Zhang

**Affiliations:** 1 Research Center for Environmental Ecology and Engineering, School of Environmental Ecology and Biological Engineering, Wuhan Institute of Technology, Wuhan, China; 2 State Key Laboratory of Marine Environmental Science, Fujian Key Laboratory of Marine Carbon Sequestration, College of Ocean and Earth Sciences, Xiamen University, Xiamen, China; 3 Department of Ocean Science, The Hong Kong University of Science and Technology, Hong Kong, China; 4 Institute of Marine Science and Technology, Shandong University, Qingdao, China; 5 Key Laboratory of Marine Genetic Resources, Third Institute of Oceanography, Ministry of Natural Resources, Xiamen, China; 6 Marine Science and Engineering Guangdong Laboratory (Zhuhai), Zhuhai, Guangdong, China; University of Miami, Coral Gables, Florida, USA

**Keywords:** *in situ* deep-sea environments, stability of viral particles, viral infectivity, stability of prokaryotes, *in situ* deep-sea long-term incubation

## Abstract

Through particles sinking as well as the movement of water masses, a fraction of bacterioplankton and virioplankton can be transported vertically from the surface to the deep oceans, and display significant changes in viral–bacterial interactions. The survival and activity of the sinking prokaryotes and viruses in the deep-sea environment is crucial for our understanding of deep-sea ecosystems and biogeochemical cycles. However, due to the substantial challenge involved *in situ* deep-sea incubation, the effect of the deep-sea environments on the ecology of surface prokaryotes and viruses is poorly studied. To fill this knowledge gap, we used an *in situ* deep-sea long-term incubation device to examine the effect of the natural deep-sea environment on the stability and activity of four viruses and their hosts (*Prochlorococcus*, *Synechococcus*, and heterotrophic bacteria) isolated from the surface ocean. Our results showed that viral particles had still not decayed completely after *in situ* incubation for 1 year, with an average retention rate of 5.69% ± 5.13% (ranging from 1.25% to 13.06%) for infectivity and 68.11% ± 40.50% (ranging from 8.55% to 99.08%) for particles. This suggests that surface viruses probably retain long-term infectivity after sinking and may influence deep-sea microbial populations in terms of activity, function, diversity, and community structure through viral–bacterial interactions and ultimately affect deep-sea biogeochemical cycles.

**IMPORTANCE:**

The survival of the sinking prokaryotes and viruses in the deep-sea environment is crucial for deep-sea ecosystems and biogeochemical cycles. Through an *in situ* deep-sea long-term incubation device, our results showed that viral particles and infectivity had still not decayed completely after *in situ* incubation for 1 year. This suggests that, via infection and lysis, surface viruses with long-term infectious activity *in situ* deep-sea environments may influence deep-sea microbial populations in terms of activity, function, diversity, and community structure and ultimately affect deep-sea biogeochemical cycles, highlighting the need for additional research in this area.

## INTRODUCTION

Generally, deep seas are defined as oceanic regions with water depths greater than 1,000 m and high hydrostatic pressures (HHPs) above 10 MPa (1). They occupy more than 80% of the global oceanic volume and are a major habitat for microbes on earth (2). In these areas, the lack of light, low temperatures (~2–3°C), and HHPs affect the deep-sea microbial ecosystem by altering microbial growth and metabolic rate (3, 4). Due to the low nutrients and energy supply, the food web of these habitats depends mainly on particulate organic matter (POM) that sinks down from the photic zone via “biological pump” (BP) (5). Microbial cells and viral particles usually adsorb on POMs and sink with them into the deep seas (6, 7). In addition, they are transported vertically by internal waves, mesoscale eddies, and so on (8 – 11). Therefore, a fraction of microbial cells and viral particles found in the deep oceans originate from the surface (12).

Previous studies indicated that prokaryotes and viruses are the two main drivers that regulate the biogeochemical cycle in the deep-sea environments (13, 14). Prokaryotes regulate the efficiency of the BP through disaggregation and remineralization of sinking POM (15, 16). Additionally, they continuously transform labile dissolved organic matter (DOM) to recalcitrant DOM in the deep-sea environments via the “microbial carbon pump” (17). As the most abundant life form in global oceans (18, 19), viruses are responsible for high bacterial mortality and reduce carbon flow to higher trophic levels by channeling POM (host cells) into DOM (e.g., nucleic acid and protein) (20 – 22). Subsequently, DOM resulted from viral lysis promotes microbial growth, affecting the biogeochemical cycle (20). A recent study also showed that the characteristics of viral–bacterial interactions in surface waters changed dramatically after these organisms were transplanted into deep-sea waters (23), indicating that prokaryotes and viruses that sink from the upper oceans into deep seas may be important factors in shaping the activity, function, diversity, and community structures of deep-sea microbial communities (24, 25). Therefore, whether sinking prokaryotes and viruses can survive in deep-sea environments could be crucial for deep-sea ecosystems and biogeochemical cycles (12, 22).

HHP is one of the most essential characteristics of a deep-sea environment, and it has been found to exert significant influences on prokaryotes and viruses. For example, a prior study reported that bacterial production rates were severely underestimated in decompressed pelagic water samples (26). HHP has also been confirmed to be an important factor that induces a significant reduction in viral infectivity (27 – 29). Interestingly, a recent study showed that viral particles retain infectivity in long-term simulated deep-sea environments (30), implying the potential ecological importance of viruses that sink from the surface to the deep seas. However, due to the substantial challenge involved *in situ* deep-sea incubation, the impact of *in situ* deep-sea environments on the survival of surface viruses and their hosts is poorly understood.

To address how surface viruses and their hosts respond to *in situ* deep-sea environments and their potential roles in the ecology and biogeochemical cycles of deep-sea ecosystems, we performed a series of incubation experiments with four isolated viruses and their hosts *in situ* deep-sea environments. Specifically, an *in situ* deep-sea long-term incubation device was used to examine the effect of a natural deep-sea environment on the activity of viruses and on the stability of viral particles and their hosts. Our results showed that viral particles and infectivity had not decayed completely after *in situ* incubation for 1 year. This suggests that surface viruses probably retain long-term infectivity after sinking and may influence viral–bacterial interactions and biogeochemical cycles in deep-sea environments.

## RESULTS

### The stability of *Prochlorococcus*, *Synechococcus*, and heterotrophic bacterial isolates

**Fig 1:**
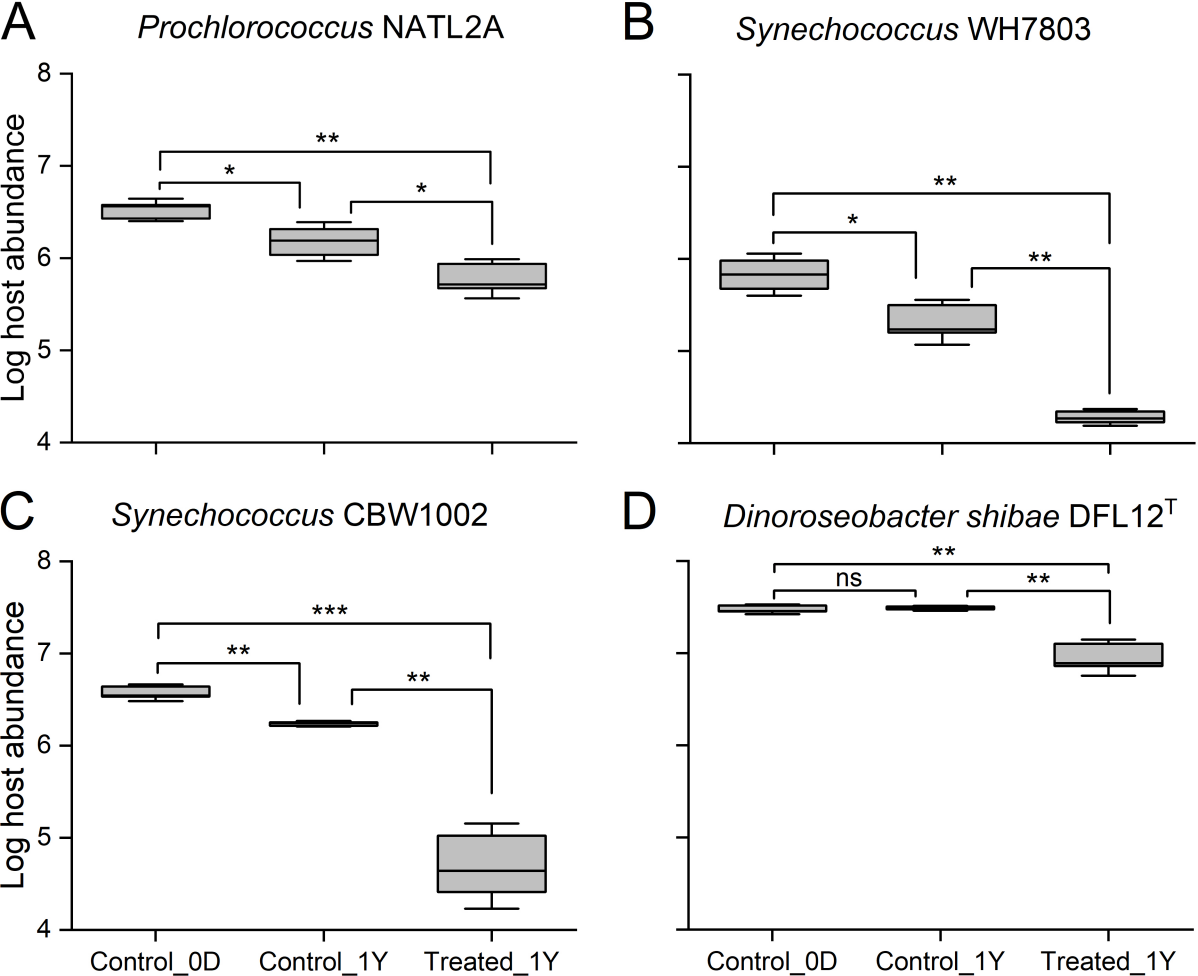
Decay of the three strains of cyanobacteria: *Prochlorococcus* NATL2A (**A**), *Synechococcus* WH7803 (**B**), and CBW1002 (**C**) and one strain of heterotrophic bacteria: *Dinoroseobacter shibae* DFL12^T^ (**D**), after 1 year of incubation under atmospheric pressure, 4°C and dark conditions (Control_1Y) and in an *in situ* deep-sea environment (Treated_1Y). Control_0D: the samples collected at the beginning of the incubations. Error bars indicate the SDs calculated from triplicate samples. ns, no significant difference; *P < 0.05; **P < 0.01; ***P < 0.001.

To explore the impact of *in situ* deep-sea environment on microbial cells, *Prochlorococcus* NATL2A, *Synechococcus* WH7803, *Synechococcus* CBW1002, and *Dinoroseobacter shibae* DFL12^T^ were incubated in an *in situ* deep-sea device at a 3,500 m depth for more than 1 year (deep-sea incubation) and in laboratory (control incubation). After incubation, the abundance of the microbial isolates in the control changed markedly (Fig. 1). The abundances of *Prochlorococcus* NATL2A, *Synechococcus* WH7803, and *Synechococcus* CBW1002 decreased significantly from 3.33 ± 0.59 × 10^6^, 6.72 ± 2.41 × 10^5^, and 3.73 ± 0.54 × 10^6^ cells mL^–1^ to 1.51 ± 0.49 × 10^6^, 2.04 ± 0.86 × 10^5^, and 1.72 ± 0.08 × 10^6^ cells mL^–1^, respectively. Interestingly, *Dinoroseobacter shibae* DFL12^T^ abundance slightly increased from 2.86 ± 0.03 × 10^7^ to 3.09 ± 0.17 × 10^7^ cells mL^–1^, although the difference was not statistically significant. The results showed that *Synechococcus* WH7803 was most sensitive to decay in the control incubation among the four prokaryotic cells, showing a 69.66% decrease in abundance, while *Dinoroseobacter shibae* DFL12^T^ had a strong resistance to decay in the control environment.

In deep-sea incubations, a more significant decay effect was observed on the four prokaryotic populations (Fig. 1). For example, the *Prochlorococcus* NATL2A abundance decreased from 3.33 ± 0.59 × 10^6^ to 5.96 ± 2.14 × 10^5^ cells mL^–1^, and *Synechococcus* WH7803 abundance decreased from 6.72 ± 2.41 × 10^5^ to 1.90 ± 0.27 × 10^4^ cells mL^–1^. In addition, the abundance of the heterotrophic bacterium *Dinoroseobacter shibae* DFL12^T^ displayed the smallest reduction of 68.81%, which was similar to the result in the control incubation, showing that it had the greatest resistance to decay in our study. *Synechococcus* CBW1002, as the prokaryotic cell that was most sensitive to decay, showed the greatest reduction, more than 98% during deep-sea incubation.

### The stability of viral particles

**Fig 2:**
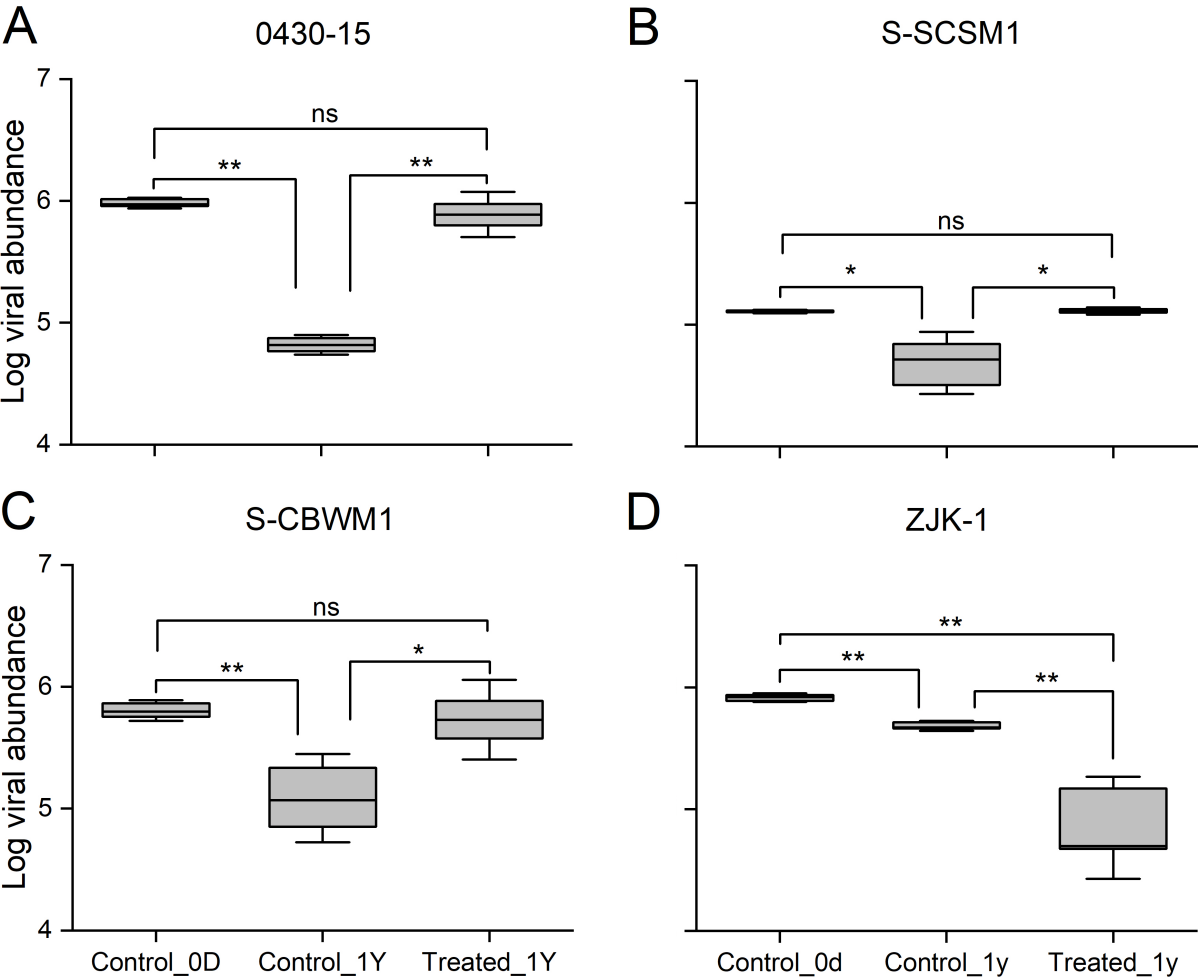
Decay of one isolated *Prochlorococcus* virus: 0430-15 (**A**), two isolated *Synechococcus* viruses: S-SCSM1 (**B**) and S-CBWM1 (**C**), and one isolated heterotrophic bacterial virus: ZJK-1 (**D**), after 1 year of incubation under atmospheric pressure, 4°C and dark conditions (Control_1Y) and in an *in situ* deep-sea environment (Treated_1Y). Control_0D: the samples collected at the beginning of the incubations. Error bars indicate the SDs calculated from triplicate samples. ns, no significant difference; *P < 0.05; **P < 0.01; ***P < 0.001.

As shown in Fig. 2, the abundance of the four viruses (0430-15, S-SCSM1, S-CBWM1, and ZJK-1) was reduced significantly in the control incubation. The *Prochlorococcus* virus 0430-15 almost completely disappeared after 1 year of incubation, and the reduction reached 93.10% (from 9.57 ± 0.65 × 10^5^ to 6.60 ± 0.82 × 10^4^ viruses mL^–1^). The decline in the two *Synechococcus* viruses was similar. The control incubation resulted in a 62.17% reduction in S-SCSM1 (from 1.28 ± 0.29 × 10^5^ to 4.85 ± 1.87 × 10^4^ viruses mL^–1^) and a 75.03% reduction in S-CBWM1 (from 6.38 ± 0.84 × 10^5^ to 1.59 ± 0.74 × 10^5^ viruses mL^–1^). However, ZJK-1 seemed more resistant to decay, showing the lowest reduction of 41.54% (from 8.23 ± 0.44 × 10^5^ to 4.81 ± 0.31 × 10^5^ viruses mL^–1^).

In contrast, during deep-sea incubation, the highest decay occurred in the heterotrophic bacterial virus ZJK-1, at 91.45% (Fig. 2). Intermediate levels of decay of 19.30% and 15.88% were observed for the *Prochlorococcus* virus 0430-15 and *Synechococcus* virus S-CBWM1, respectively. These two viruses displayed a relatively strong resistance to decay in both deep-sea and control incubations. Furthermore, the *Synechococcus* virus S-SCSM1 was found to be most resistant to decay. Its abundance after deep-sea incubation did not differ significantly from that at the beginning of incubation.

### Viral infectivity

**Fig 3:**
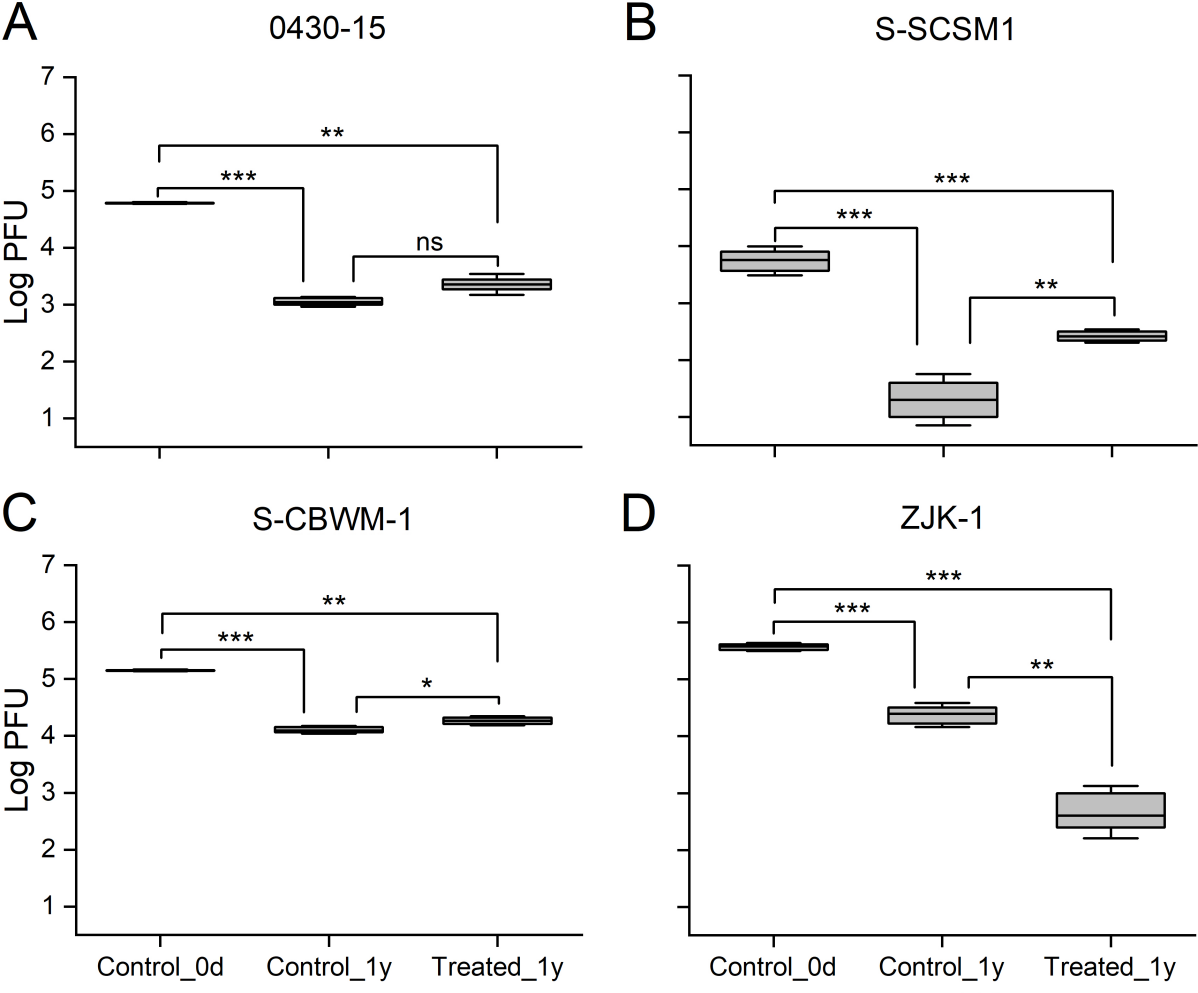
Inactivation of one isolated *Prochlorococcus* virus: 0430–15 (**A**), two isolated *Synechococcus* viruses: S-SCSM1 (**B**) and S-CBWM1 (**C**), and one isolated heterotrophic bacterial virus: ZJK-1 (**D**), after 1 year of incubation under atmospheric pressure, 4°C and dark conditions (Control_1Y) and in an *in situ* deep-sea environment (Treated_1Y). Control_0D: the samples collected at the beginning of the incubations. Error bars indicate the SDs calculated from triplicate samples. ns, no significant difference; *P < 0.05; **P < 0.01; ***P < 0.001.

Similar to the results of the decay of viral particles, the infectivity of the four viruses decreased during both deep-sea and control incubations (Fig. 3). Specifically, the control incubation caused a decline of 0430-15, S-SCSM1, and ZJK-1 infectivity from 6.10 ± 0.14 × 10^4^, 5.53 ± 2.15 × 10^4^, and 3.72 ± 0.40 × 10^5^ PFU mL^–1^ to 1.13 ± 0.15 × 10^3^, 2.00 ± 1.53 × 10^1^, and 2.38 ± 0.76 × 10^4^ PFU mL^–1^, respectively. Similarly, with reductions of more than 96%, 95%, and 99%, the infectivity of 0430-15, S-SCSM1, and ZJK-1 were nearly completely lost during deep-sea incubation. The minimum inactivation was observed in S-CBWM1, with a reduction of 91.03% in the control incubation and 86.94% in the deep-sea incubation; thus, it displayed the strongest resistance to inactivation among the four viruses. Furthermore, the three viruses, 0430-15, S-SCSM1, and S-CBWM1 showed similar patterns; each infectivity was more fully preserved during deep-sea incubation than during control incubation. In contrast, ZJK-1, a heterotrophic bacterial virus, was almost completely inactivated during deep-sea incubation while maintained ~6% of its infectivity during control incubation.

## DISCUSSION

In oceans, some viral particles ultimately decay and enter the DOM pool (31). Their stability is impacted by complex biotic and abiotic factors, such as solar radiation, temperature, pressure, and extracellular enzymes (3, 32 – 35), and is also related to their structural and physiological characteristics (31, 33). In our experimental design, the main differences in environmental parameters between the control and *in situ* deep-sea incubations were HHP and mobility (i.e., water exchange between the incubator and the external seawater).

### Stability of surface viral particles in a deep-sea environment

The decay of the three *Myoviridae* viruses (i.e., 0430-15, S-SCSM1, and S-CBWM1) in the deep-sea incubation was significantly less than that during control incubation. This result may be caused by different activity of extracellular enzymes (e.g., proteases and nucleases), which are important factors in destroying the structure of viral particles (36, 37), in these two incubations. The deep-sea indigenous extracellular enzymes could not pass through the 0.01 µm polycarbonate membrane to affect viral decay in deep-sea incubations. Under a similar incubation temperature (~4°C), extracellular enzymes could still have activity to promote viral decay in the control incubation with atmospheric pressure (38), while their activity might be destroyed or suppressed by HHP in the deep-sea incubations. For example, Silva et al. (39) found that HHP can cause some proteins to dissociate and thereby inactivate (39). Therefore, the denaturation of extracellular enzymes caused by HHP may weaken viral decay and allow more viral particles to be preserved *in situ* deep-sea environments.

ZJK-1, the only *Siphoviridae* virus*,* displayed minimal decay among the four viruses in the control incubation conducted under atmospheric pressure (Fig. 2). Consistent with previous studies, *Siphoviridae* showed the most resistance to decay against environmental factors under atmospheric pressure, which may accompany a potential association with their flexible tails (40 – 42). Interestingly, ZJK-1 viral particles were most sensitive to *in situ* deep-sea environments (Fig. 2). A prior study indicated that viral decay was positively correlated with the internal pressure of the viral capsid caused by the density of the packaged genome (*ρ*_pack_) (33). Therefore, viruses that infect heterotrophic bacteria were considered to have relatively high *ρ*_pack_ values (35), which is consistent with this study, that is, the highest *ρ*_pack_ was observed in ZJK-1 among the four viruses (Table S1), and HHP may have amplified this effect. In addition, the ZJK-1 virus is a *Siphoviridae* and has a long tail, and the other three viruses that exhibited relatively high resistance to decay under HHP are all *Myoviridae*. Solomon et al. demonstrated that HHP produced distinctive alterations in the viral tail structure (43), suggesting that the sophisticated structure of long viral tails may increase their sensitivity to HHP (44). Herein, we speculate that the destructive effect of the *in situ* deep-sea environment on viruses may be closely related to the morphology of the viral tail.

The *in situ* deep-sea environments seemed to be minimally destructive on the viral capsids and internal nucleic acids of S-SCSM1, and a large number of their particles were preserved, which may be indirectly explained by some underlying mechanisms in previous studies. Viral capsids are generally permeable toward water and salt ions (45, 46), indicating that osmotic equilibrium could be maintained between the internal capsid and the external environment. A study showed that the permeability of some viruses was significantly increased by the dissociation and denaturation of key viral capsid proteins (47). Therefore, the decay of viral particles in the deep-sea incubation may be related to the difference in the permeability of their capsids because of the difference in the dissociation and denaturation effect of HHP on viral proteins. In addition, Silva et al. (48) found that the interactions of viral capsid proteins increased the stability of coat protein subunits for resisting denaturation (48). The formation of nucleoprotein complexes can increase the stability of the overall virus (49). These results indicated that the impact of HHP on viral stability may be closely related to the viral structure.

### Activity of surface viruses in a deep-sea environment

Similar to factors affecting viral stability, infectivity is also impacted by solar radiation, temperature, pressure, extracellular enzymes, and so on (34, 50). Theoretically, inactivation consistently precedes the complete decay of viral particles since if the structure of a viral particle is incomplete, the virus may not be able to complete the infection cycle (e.g., fail to bind to host cells or inject nucleic acids) (33). Therefore, compared with their stability (Fig. 2), the activity of the four viruses was much more sensitive in both incubation experiments (Fig. 3). However, the four viruses all partly maintained infectivity after 1 year of incubation. For instance, the infectivity of S-CBWM1 was greater than 13% at the end of deep-sea incubation. This indicated that surface viruses retain their ability to infect their hosts in deep seas for a long time and may continuously lyse their host during and after sinking (23). Except for ZJK-1, infectivity was higher in deep-sea incubations than in control incubations. Therefore, the infectivity of some viruses seemed to be retained to a greater degree in the deep-sea environment. This may be because fewer viral particles decayed in the deep-sea environment (Fig. 2). However, the pattern of change in the normalized viral activity ratio was consistent (Fig. S1). They were all lowest in the deep-sea incubation, followed by that at the beginning of incubation, and highest in the control incubation. This indicated that HHP has a significant negative effect on virus infectivity, which is consistent with previous studies (27 – 29). Some studies found that HHP can induce small changes in viral structures without decaying of the entire particle (51, 52). As a consequence, viral inactivation during deep-sea incubation is probably because HHP induced denaturation of some viral tail proteins that are essential for host cell attachment, preventing binding to host cells for infection (28, 53 – 56).

In addition, viruses are reported to have a wide range in intrinsic susceptibility to HHP (29, 44). After deep-sea incubation for 1 year, S-CBWM1 retained more than 13% infectivity, but the infectivity of ZJK-1 was reduced by ~99.88%, displaying a larger difference in viral inactivation. In addition, the infectivity of S-CBWM1 was most preserved in both incubations among the four viruses, indicating that S-CBWM1 has strong resistance to decay after 1 year of incubation. This also indicates that, as another *Myoviridae* virus that infects *Synechococcus*, S-CBWM1 has stronger resistance to decay in the deep-sea environment than S-SCSM1. Similarly, Oliveira et al. (57) reported that picornaviruses respond differently to inactivation caused by HHP (57). Kingsley et al. (58) suggested that compared with receptor binding mediated by the arginine-glycine-aspartate motif, picornaviruses, which use a canyon feature for receptor binding, are significantly more resistant to HHP, indicating that the mode of receptor recognition influences viral resistance to HHP (58). This may be one of the potential mechanisms for the difference in infectivity of the four viruses in our study but need further experimental verification.

### *In situ* incubation vs simulated incubation

Compared with the results of our previous study (30), it was found that the particles and infectivity of the *Synechococcus* viruses, S-SCSM1 and S-CBWM1, were retained more in the *in situ* deep-sea environments than in a simulated high-pressure incubation device. The pressurization and decompression processes are performed within relatively short periods of time in the high-pressure simulation device (e.g., in minutes), which probably results in an imbalance in HHP between the inside and outside of viral capsids, promoting the rupture and decay of viral particles in the environment. However, the slow placement and recycling of the incubation device in this study might weaken the damaging effects of a pressure difference on the viral particles during deep-sea incubation. The *in situ* deep-sea long-term incubation device allowed a couple of hours for viral particles to adjust the HHP imbalance between the inside and outside of the viral capsid by osmosis (i.e., samples slowly sinking from the sea surface to the deep sea), hence reducing shock and destruction due to a pressure difference on the viruses. It is worth noting that during the natural vertical sinking process, viruses adsorbed on particles may sink to the deep sea at a slower rate (possibly weeks to months) (59). We speculate that natural sinking may tend to further weaken the negative effect of HHP on viral stability and infectivity.

Additionally, compared with simulated incubation in a closed system, *in situ* incubation in an open system can allow small compounds and ions to pass freely, which may affect viral activity and degradation. Some organic matter was reported to confer a protective effect on viral stability during HHP (60 – 62). For example, some amino acids were reported to protect the infectivity of poliovirus from inactivation (63). As amino acids are capable of forming metal complexes, the protective effect might occur because amino acids combine with harmful metal ions to protect viral infectivity (e.g., Cu^2+^ and Hg^2+^) (64). However, Larsson et al. (47) found that two Ca^2+^ binding sites on viral capsid were pivotal in the expansion and capsid-opening process, playing a key role in viral stability (47). Zn^2+^, Fe^3+^, and Al^3+^ were also considered to have a protective effect on viral infectivity (63), but the mechanism for this protective effect is poorly understood. Nevertheless, compared with the completely closed incubation system used in our previous study (30), we speculate that small molecular substances and metal ions of size less than 0.01 µm (which freely pass through the dialysis bag used in this study) in the *in situ* deep-sea environments may protect the survival of viruses to a certain extent.

### Survival of surface prokaryotes in a deep-sea environment

Deep-sea hydrostatic pressure is one of the crucial factors affecting the growth, metabolic activity, and survival of microbial cells (4). Accordingly, we found that deep-sea incubation for 1 year increased the decay of *Prochlorococcus* NATL2A, *Synechococcus* WH7803, *Synechococcus* CBW1002, and *Dinoroseobacter shibae* DFL12^T^ (Fig. 1). Since the prokaryotic cells used in this study were isolated from the surface ocean under atmospheric pressure, the *in situ* deep-sea environment deviated far from their optimal growth environment (e.g., light, temperature, HHP, pH, redox potential, and organic/inorganic nutrition), probably impairing their survivability. One of the underlying mechanisms for the effect of HHP may be that it alters the conformation of membrane proteins on the cell surface to expose key sites to the environment; thus, the proteins are more easily damaged, resulting in losing their ability to metabolize and finally being decayed (65). For instance, HHP could impact the functional proteins responsible for transporting ions and amino acids, which promotes destabilization of the cell membrane (66, 67).

As the two main groups of autotrophic cyanobacteria, *Prochlorococcus* NATL2A appears to be more resistant to decay than *Synechococcus* WH7803 and CBW1002, showing final decay rates of 82.11% vs 97.17% and 98.68%, respectively, in deep-sea incubations. Evidence to confirm this result can be found in previous physiological and ecological studies. Rocap et al. (68) reported that some *Prochlorococcus* strains contained oligopeptide transporters in their genomes (68), indicating that *Prochlorococcus* has some potential for heterotrophic survival (69). Another study found that the natural population of *Prochlorococcus* has a greater capacity to survive than *Synechococcus* under low irradiance conditions (70). These results suggested that *Prochlorococcus* may have a higher potential to survive in deep-sea incubations than *Synechococcus*, although the cell activity was not measured in our study. Furthermore, the *Dinoroseobacter shibae* DFL12^T^ showed the strongest environmental adaptability against decay in our *in situ* deep-sea incubation among the four prokaryotic cells. Heterotrophic bacteria do not depend on light for metabolism and growth. Therefore, they are theoretically more adapted to dark deep-sea environments than autotrophic cyanobacteria, although they are all affected by HHP.

### Ecological significance of sinking surface viruses

Previous studies have indicated that viral structures and infectivity can be maintained for periods of decades to a century for certain viruses in sediments (71, 72). Some proteins sustain their molecular identities in the deep oceans on decadal to centennial timescales (73, 74). Therefore, Yang et al. (75) suggested that the turnover time of viral particles in a deep-sea environment is likely to be on the time scale of years (75). Our study showed that the resident time of surface viruses exceeded 1 year in an *in situ* deep-sea environment, indirectly supporting the above speculation on the long viral turnover time. It is worth noting that viruses still have partial infectivity after incubation for 1 year. Although the underlying mechanisms of the long-term preservation of both viral stability and activity in deep-sea environments are poorly understood (74), via infection and lysis, surface viruses with long-term infectious activity *in situ* deep-sea environments may influence deep-sea microbial populations in terms of activity, function, diversity, and community structure and ultimately affect deep-sea biogeochemical cycles, and these topics require further attention.

## MATERIALS AND METHODS

### Viruses, their hosts, and sample preparation

Four phages that were isolated from surface waters and their hosts were used in our incubation experiments (Table S1). They included one *Prochlorococcus* virus, 0430-15, and its host, *Prochlorococcus* NATL2A (76); two *Synechococcus* viruses, S-SCSM1 (77) and S-CBWM1 (78), as well as their hosts, *Synechococcus* WH7803 and CBW1002, respectively; and one heterotrophic bacterial virus, ZJK-1, and its host, *Dinoroseobacter shibae* DFL12^T^ (79, 80). The culture for *Prochlorococcus* was maintained in Pro99 medium under constant light flux (25 µmol Q m^−2^ s^−1^) at 22°C. Autotrophic *Synechococcus* were incubated in an SN medium with 15‰ salinity for CB1002 and A + medium for WH7803 at 22°C under continuous light (20 µmol photons m^−2^ s^−1^). Heterotrophic *Dinoroseobacter shibae* DFL12^T^ was incubated in a rich organic medium at 25°C with agitation at 180 rpm min^−1^.

During the preparation of the incubation system, to make the initial viral abundance of each treatment close to that of the *in situ* condition, each virus sample was diluted with seawater (~3,000 m depth) that was filtered through a 30 kDa membrane (Polycarbonate, Millipore, Billerica, MA, USA), and the final concentration was adjusted to 10^5^–10^6^ viruses mL^−1^ by flow cytometry determination (81, 82). Then, each diluted sample was packaged in a dialysis bag (in triplicate) and placed in a 50 mL aseptic tube filled with deep-sea water. The nozzle of the aseptic tube was sealed with a 0.01 µm polycarbonate membrane. To maintain material exchange between the inside of the aseptic tube and the *in situ* deep-sea water, several small holes were made on the aseptic tube cover.

**Fig 4:**
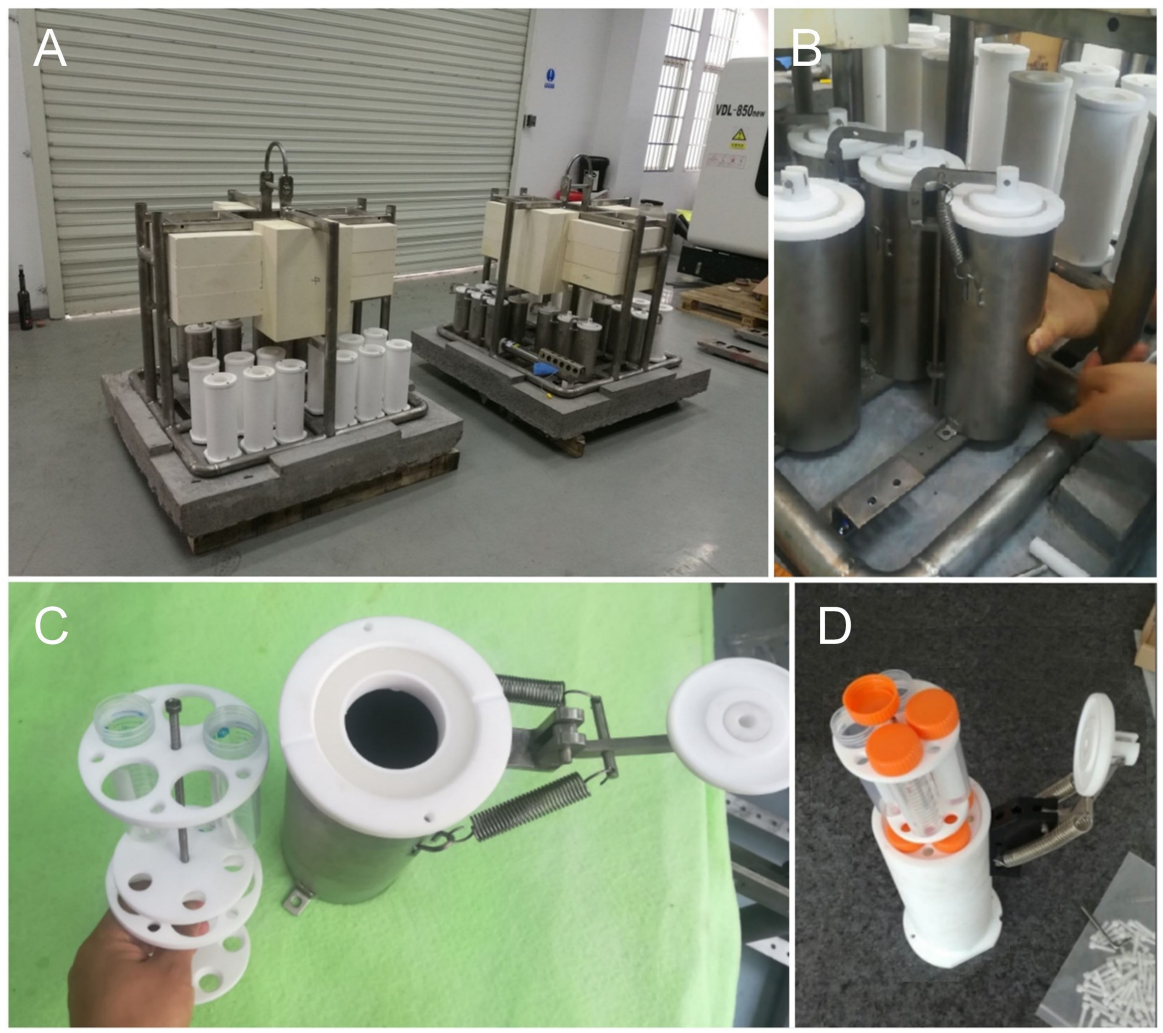
The *in situ* long-term deep-sea incubation device (**A**) and the details of device accessories (**B–D**). It was designed and manufactured by the Third Institute of Oceanography of the Ministry of Natural Resources, China, and used in this study for 1-year incubations of four isolated viruses and their hosts.

During the *R/V XiangYangHong 03* cruise, the treatment group consisting of the prepared viruses and host samples (marked as Treated_1Y) were incubated at a 3,500 m depth (~35 MPa HHP) at Station MIES1 (116.06°E, 18.45°N) located in the northern basin of the South China Sea for 1 year (from 19 June 2017 to 8 July 2018) using the deep-sea microbial *in situ* enrichment culture system in an *in situ* deep-sea long-term incubation device. At the same time, the other group of viruses and host samples were incubated for 1 year in an enclosed system under atmospheric pressure, 4°C, and dark conditions without any disturbance or material exchange (marked as control_1Y). Before the onset of the experiments (19 June 2017), samples were collected from all incubations and marked as control_0D. To reduce sample cross-contamination risk, the lid-sealed aseptic tubes containing virus and host samples for *in situ* deep-sea incubation were separated into different culture chambers (Fig. 4A and B). During *in situ* incubation, the cover for the culture chamber was left in an open state to allow for water exchange between the incubator and the external seawater (Fig. 4C and D).

### Measurement of viral and host abundance

After 384 days of incubation, subsamples (1 mL) were taken from each dialysis bag to determine viral and host abundances. They were fixed with glutaraldehyde (0.5% final concentration) and incubated at 4°C for 15 min in the dark. After flash-freezing in liquid nitrogen, the samples were stored at –80°C before analysis. By analyzing flow cytometry (Epics Altra II, Beckman Coulter) scatter plots for side scatter versus red fluorescence and for orange fluorescence versus red fluorescence, the *Prochlorococcus* and *Synechococcus* abundances could be directly determined (83). After cells were stained with 1.0 × 10^–4^ SYBR Green I (vol/vol, final concentration of the stock solution, Molecular Probes) and then incubated for 15 min in the dark, heterotrophic bacterial abundance was determined by flow cytometry with scatter diagrams of side scatter versus green fluorescence (83). In addition, subsamples were diluted with Tris-EDTA buffer (pH 8.0; Sigma, St. Louis, MO, USA), stained with 5.0 × 10^–5^ SYBR Green I (vol/vol, final concentration of the stock solution), and incubated at 80°C for 10 min. Then viral abundance was determined by flow cytometry with a scatter diagram of side scatter versus green fluorescence (81, 82). Finally, all data analysis was performed with FCS Express V3 software (*De Novo* Software, Los Angeles, CA, USA).

### Determination of viral infectivity

A plaque assay method was used to determine viral infectivity. In this work, viral infectivity is expressed as the number of plaque-forming units per milliliter (PFU mL^–1^) (84). Specifically, viral samples were serially diluted 10-fold with sterile media. Then, each diluted sample was separately mixed with host cultures to allow the viruses to adsorb onto their host cells. The subsamples were mixed with soft agar (0.5% final concentration) and spread evenly on agar plates (1% final concentration) to visualize plaques. Finally, the viral infectivity in each sample was determined by counting the number of plaques after 1–2 days for heterotrophic bacteria and after 1–2 weeks for cyanobacteria (Fig. S2).

### Statistical analysis

In this study, one-way analysis of variance was performed to assess the statistical significance among groups of different treatments in SPSS 19 software (SPSS Inc., Chicago, IL, USA). Differences were considered significant at a *P* value <0.05 (Table S2).

## SUPPLEMENTAL MATERIAL

Supplemental information spectrum.04534-22-s0001.docx Fig. S1 and S2 and Tables S1 and S2.

ASM does not own the copyrights to Supplemental Material that may be linked to, or accessed through, an article. The authors have granted ASM a non-exclusive, world-wide license to publish the Supplemental Material files. Please contact the corresponding author directly for reuse.
