# Supplementary material for: Survival of surface bacteriophages and their hosts in in situ deep-sea environments
Source: Microbiol Spectr. 2023 Dec 5;12(1):e04534-22. doi: 10.1128/spectrum.04534-22 (PMC10783000; doi:10.1128/spectrum.04534-22)
Supplement: Supplemental information — Fig. S1 and S2 and Tables S1 and S2. [file spectrum.04534-22-s0001.docx]

**Survival of surface bacteriophages and their hosts in *in situ* deep-sea environments (Supplement information)**

Wei Wei^a,b,†^, Yuan Tian^b,†^, Lanlan Cai^c^, Yongle Xu^d^, Xilin Xiao^b^, Qiong Wang^b^, Haowen Wang^b^, Chunming Dong^e^, Zongze Shao^e^, Nianzhi Jiao^b^, Rui Zhang^b,f,*^

^a^ Research Center for Environmental Ecology and Engineering, School of Environmental Ecology and Biological Engineering, Wuhan Institute of Technology, Wuhan 430205, PR China

^b^ State Key Laboratory of Marine Environmental Science, Fujian Key Laboratory of Marine Carbon Sequestration, College of Ocean and Earth Sciences, Xiamen University, Xiamen 361102, PR China

^c^ Department of Ocean Science, The Hong Kong University of Science and Technology, Hong Kong, PR China

^d^ Institute of Marine Science and Technology, Shandong University, Qingdao 266000, PR China

^e^ Key Laboratory of Marine Genetic Resources, Third Institute of Oceanography, Ministry of Natural Resources, Xiamen 361005, PR China

^f^ Marine Science and Engineering Guangdong Laboratory (Zhuhai), Zhuhai, Guangdong, PR China

^†^Wei Wei and Yuan Tian contributed equally to this work.

**^*^**Corresponding author: Rui Zhang ([ruizhang@xmu.edu.cn](mailto:ruizhang@xmu.edu.cn)).

The authors declare no conflict of interest.


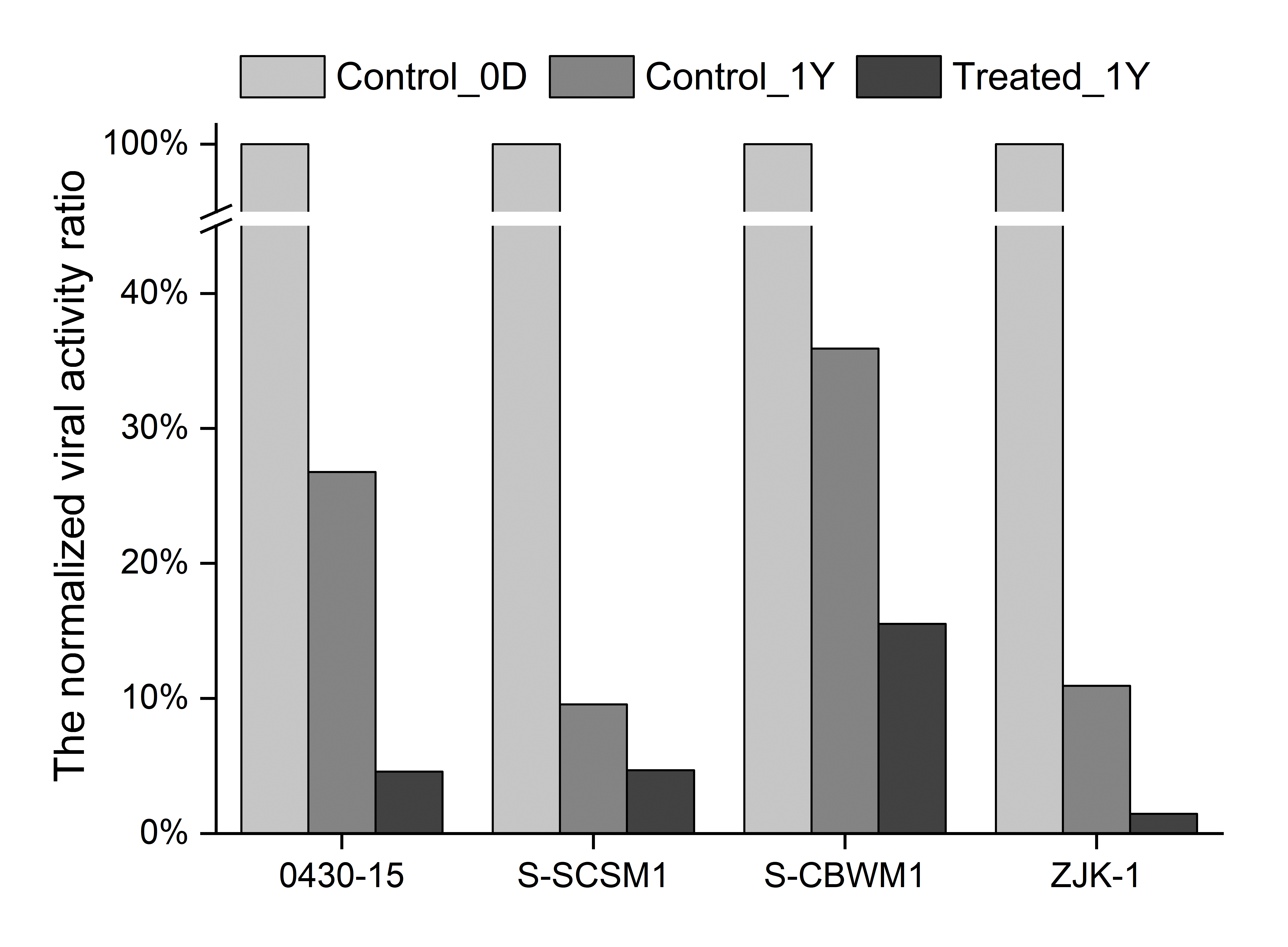


**FIG S1** The normalized viral activity ratio (i.e., $a \times\frac{\mathrm{PFU}}{\mathrm{VA}} \times100\%$, where a = $\frac{\mathrm{VA}}{\mathrm{PFU}}$ in Control_0D as a constant) of one isolated *Prochlorococcus* virus: 0430-15, two isolated *Synechococcus* viruses: S-SCSM1 and S-CBWM1, and one isolated heterotrophic bacterial virus: ZJK-1, after one year of incubation under atmospheric pressure, 4°C and dark conditions (Control_1Y) and in an *in situ* deep-sea environment (Treated_1Y). Control_0D: the samples collected at the beginning of the incubations; VA: viral abundance.


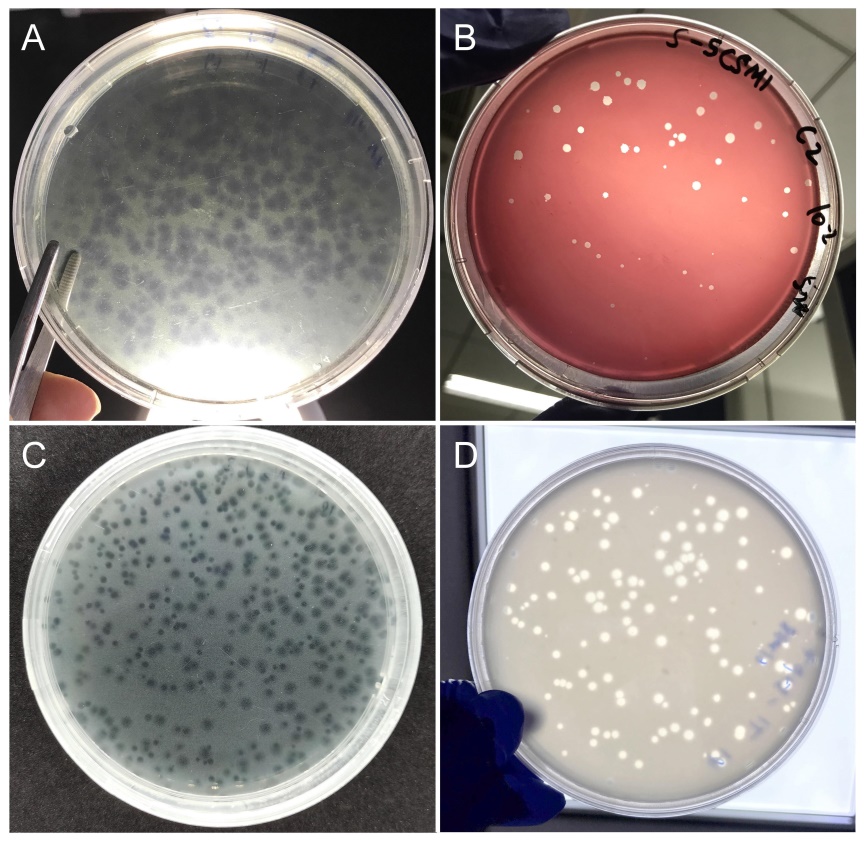


**FIG S2** Plate images of the plaques for 0430-15 infecting *Prochlorococcus* NATL 2A (A), S-SCSM1 infecting *Synechococcus* WH7803 (B), S-CBWM1 infecting *Synechococcus* CBW1002 (C) and ZJK-1 infecting *Dinoroseobacter shibae* DFL12^T^ (D), respectively.

| **TABLE S1.** The four isolated viruses that infect autotrophic and heterotrophic prokaryotes used in this study. | | | | | | | |  |  |  |
| --- | --- | --- | --- | --- | --- | --- | --- | --- | --- | --- |
| Phage nomenclature | Host strain | Classification | Morphology | Capsid size (nm) | Tail size (nm) | Capsid volume (nm^3^) | Genome size (bp) | ρ_pack_ (bp/nm^3^) | Isolation place |  |
| 0430-15 | *Prochlorococcus* NATL 2A | Unclassified | Myovirus | 80 |  | 267,947 | 119,110 | 0.44 | Yongxing Island, China | Wang et al. unpublished data |
| S-SCSM1 | *Synechococcus* WH7803 | T4-like phage | Myovirus | 100 | 173 × 11 | 463,247 | 228,827 | 0.49 | South China Sea | Wang et al. (2022) |
| S-CBWM1 | *Synechococcus* CBW1002 | Unclassified | Myovirus | 76 | 170 × 22 | 229,847 | 139,069 | 0.65 | Baltimore Inner Harbor, The United States | Xu et al. (2018) |
| ZJK-1 | *Dinoroseobacter shibae* DFL12^T^ | Unclassified | Siphovirus | 62 × 45 | 175 | 98,557 | 79,534 | 0.81 | Pearl River Estuary, Guangdong, China | Lu et al. unpublished data |

**REFERENCES**

<Wang> Q, [Cai](https://journals.asm.org/doi/10.1128/spectrum.02367-22#con2) L, [Zhang](https://journals.asm.org/doi/10.1128/spectrum.02367-22#con3) R, [Wei](https://journals.asm.org/doi/10.1128/spectrum.02367-22#con4) S, [Li](https://journals.asm.org/doi/10.1128/spectrum.02367-22#con5) F, [Liu](https://journals.asm.org/doi/10.1128/spectrum.02367-22#con6) Y, [Xu](https://journals.asm.org/doi/10.1128/spectrum.02367-22#con7) Y. 2022. A unique set of auxiliary metabolic genes found in an isolated cyanophage sheds new light on marine phage-host interactions. *Microbiol Spectr* 10: e02367-22. <https://doi.org/10.1128/spectrum.02367-22>.

Xu Y, Zhang R, Wang N, Cai L, Tong Y, Sun Q, Chen F, Jiao N. 2018. Novel phage-host interactions and evolution as revealed by a cyanomyovirus isolated from an estuarine environment. *Environ Microbiol* 20:2974–2989. https://doi.org/10.1111/1462-2920.14326.

| **TABLE S2.** The *p* values of the one-way ANOVA analysis among groups of different treatments for the four isolated viruses and their hosts in this study. | | | | | | | | | |
| --- | --- | --- | --- | --- | --- | --- | --- | --- | --- |
| **Host abundance** | | *Prochlorococcus* NATL 2A | | *Synechococcus* WH7803 | | *Synechococcus* CBW1002 | | *Dinoroseobacter shibae* DFL12^T^ | |
|  |  | Control_1Y | Treated-1Y | Control_1Y | Treated-1Y | Control_1Y | Treated-1Y | Control_1Y | Treated-1Y |
| *Prochlorococcus* NATL 2A | Control_0D | 0.031 | 0.003 |  |  |  |  |  |  |
|  | Control_1Y |  | 0.024 |  |  |  |  |  |  |
| *Synechococcus* WH7803 | Control_0D |  |  | 0.016 | 0.004 |  |  |  |  |
|  | Control_1Y |  |  |  | 0.004 |  |  |  |  |
| *Synechococcus* CBW1002 | Control_0D |  |  |  |  | 0.006 | 0.000 |  |  |
|  | Control_1Y |  |  |  |  |  | 0.001 |  |  |
| *Dinoroseobacter shibae* DFL12^T^ | Control_0D |  |  |  |  |  |  | 0.647 | 0.003 |
|  | Control_1Y |  |  |  |  |  |  |  | 0.002 |
| **Viral abundance** | | 0430-15 | | S-SCSM1 | | S-CBWM1 | | ZJK-1 | |
|  |  | Control_1Y | Treated-1Y | Control_1Y | Treated-1Y | Control_1Y | Treated-1Y | Control_1Y | Treated-1Y |
| 0430-15 | Control_0D | 0.000 | 0.477 |  |  |  |  |  |  |
|  | Control_1Y |  | 0.001 |  |  |  |  |  |  |
| S-SCSM1 | Control_0D |  |  | 0.032 | 0.232 |  |  |  |  |
|  | Control_1Y |  |  |  | 0.029 |  |  |  |  |
| S-CBWM1 | Control_0D |  |  |  |  | 0.007 | 0.714 |  |  |
|  | Control_1Y |  |  |  |  |  | 0.021 |  |  |
| ZJK-1 | Control_0D |  |  |  |  |  |  | 0.002 | 0.003 |
|  | Control_1Y |  |  |  |  |  |  |  | 0.007 |
| **PFU** | | 0430-15 | | S-SCSM1 | | S-CBWM1 | | ZJK-1 | |
|  |  | Control_1Y | Treated-1Y | Control_1Y | Treated-1Y | Control_1Y | Treated-1Y | Control_1Y | Treated-1Y |
| 0430-15 | Control_0D | 0.000 | 0.005 |  |  |  |  |  |  |
|  | Control_1Y |  | 0.142 |  |  |  |  |  |  |
| S-SCSM1 | Control_0D |  |  | 0.000 | 0.000 |  |  |  |  |
|  | Control_1Y |  |  |  | 0.003 |  |  |  |  |
| S-CBWM1 | Control_0D |  |  |  |  | 0.000 | 0.003 |  |  |
|  | Control_1Y |  |  |  |  |  | 0.016 |  |  |
| ZJK-1 | Control_0D |  |  |  |  |  |  | 0.000 | 0.000 |
|  | Control_1Y |  |  |  |  |  |  |  | 0.004 |
